# A Pilot Multi-Omics Approach Unveils Strong Immune Activation in the First Ten Days of Life in Extremely Preterm Infants

**DOI:** 10.3390/metabo15100659

**Published:** 2025-10-07

**Authors:** Laura Burgess, Eva Caamaño Gutiérrez, Brian F. Flanagan, Duncan Alexander Sylvestre, Carolyn M. Slupsky, Mark A. Turner, Colin Morgan

**Affiliations:** 1 Department of Neonatology, Liverpool Women’s Hospital, Crown Street, Liverpool L8 7SS, UK; laura.burgess@lwh.nhs.uk (L.B.); mark.turner@liverpool.ac.uk (M.A.T.); 2 Computational Biology Facility, LIV-SRF, University of Liverpool, Liverpool L69 7ZB, UK; eva.caamano-gutierrez@liverpool.ac.uk; 3 Department of Women’s and Children’s Health, Institute of Translational Medicine, University of Liverpool, Alder Hey Children’s NHS Foundation Trust Hospital, Eaton Road, Liverpool L12 2AP, UK; fla1@liverpool.ac.uk; 4 Department of Food Science and Technology, University of California Davis, One Shields Ave, Davis, CA 95616, USA; dsylvestre@ucdavis.edu (D.A.S.); cslupsky@ucdavis.edu (C.M.S.); 5 Department of Nutrition, University of California Davis, One Shields Ave, Davis, CA 95616, USA

**Keywords:** transcriptomics, microarray, metabolomics, parenteral nutrition, immunonutrition, preterm, neonate

## Abstract

**Background:** Very preterm infants (VPIs) are born with an undeveloped immune system and are more susceptible to infection. Acquired immune responses must develop in a complex nutritional and metabolic environment as these babies transition from parenteral to enteral nutrition. We explored the feasibility of a multi-omics approach to investigate the changes in metabolic and molecular profiles between day 3 and day 10 of life. **Methods:** Blood and plasma samples were collected at day 3 and day 10 of life from eight infants born <29 weeks’ gestation and used to perform microarray transcriptomics and ^1^H NMR metabolomics. All data were analysed using univariate statistics and mapped to biological pathways and molecular functions using an assortment of databases. **Results:** We found 1185 genes differentially expressed. The expression patterns found mapped to different immune function, maturation, and development pathways as well as providing mechanistic insights into metabolic changes, notably the downregulation of the metallothionein pathways. We found five metabolites that presented significant differential abundance. These linked to sugar and fat metabolic pathways, known to be altered in the preterm infants. **Conclusions:** We show that a multi-omics approach is feasible in VPIs and can identify simultaneous changes in the complex metabolic processes and immune adaptation that occur in the first ten days of life.

## 1. Introduction

The rapid physiological adaptation required by the cardiorespiratory system following very preterm infant delivery is well recognised. The slower metabolic adaptation that takes place over the first two weeks of life is less well understood and incorporates the transition from transplacental metabolism and transfer of nutrients to full enteral feeding via a variable period of parenteral nutrition (PN) dependence [1]. The period of PN dependence is associated with both birthweight and gestation but dominates nutrient provision in the first 10 days of life in infants born <29 weeks’ gestation [2,3,4]. This period of PN dependence coincides with a key period of immune system development and metabolic changes as the infant adapts to ex utero life. The potentially complex interaction between nutritional intake, metabolic adaptation, and immune system maturation lends itself to a multi-omics investigative approach.

The relationship is further complicated by the fact that administration of PN is itself a risk factor for sepsis because of the potential need for central venous catheters. The major PN macronutrient components: amino acids, glucose, and lipids, are all individually associated with sepsis. Thus, high-dose amino acids have been associated with increased risk of sepsis in both preterm [5] and term [6] infants. The latter study has identified early introduction of parenteral amino acids as the macronutrient associated with poorer outcomes [7]. In PN-dependent preterm infants, glucose load is also a risk factor for early sepsis [8]. Bacterial clearance may be impaired by both hyperglycaemia [9] and hyperlipidaemia [10]. Other mechanisms, such as changes in IGF-1:insulin:glucose metabolism and pancreatic beta-cell remodelling in the first two weeks after birth, may also be important contributing factors in glucose metabolism. Despite biologically plausible mechanisms, no differences in immune or inflammatory responses related to intravenous lipid components have been identified [11].

Conversely, there are individual parenteral nutrients that have the potential to enhance the preterm immune response. Immunonutrition in preterm infants [12] often focuses on enteral nutrition, but parenteral elements including amino acid (arginine, glutamine) [13] and omega-3 fatty acids [14], must also be considered. Some immunonutrients have a wide range of bioactivity that may lead to either unintended benefits or risks to metabolic process outside the immune or inflammatory responses. For example, arginine is not only an immunomodulatory agent; it is also the main substrate for nitric oxide synthesis, a key intermediate in the urea cycle, a potent insulin secretagogue, a precursor to polyamine and proline synthesis, and comprises 14% of cellular protein amino acids [15]. Given that many immunonutrients in PN are modifiable, it is logical to work towards a PN composition and regimen that optimises immune system development. Multi-omics analysis offers an inclusive approach to investigating multiple biological mechanisms operating simultaneously.

All infants are born with an immature immune system which must adapt from life in the protective uterine environment to survival in a microbiologically hostile external world. In the immediate postnatal period, a newborn relies primarily on innate immune responses, implemented by immune cells such as neutrophils and macrophages, and maternal antibodies transferred during pregnancy or in colostrum, to provide protection against infection [16] (Tsafaras 2020). Although B- and T- lymphocytes are present in the blood at birth, the adaptive immune system is widely considered to be functionally immature, and it is generally accepted that even in term infants the adaptive immune system must undergo a transition from being largely unresponsive or tolerant to becoming mature and functionally active, including the formation of additional lymphoid tissue [17] (Yu 2018). These changes are influenced by exogenous antigens such as environmental pathogens, infant diet [18] (Atyeo 2021), or metabolism [19] (Holm 2021). For example, the development of gut-associated lymphoid tissue (GALT) is dependent on exposure to environmental antigens and plays a role in shaping the infant’s immune system during this time.

Very preterm infants (VPIs), born at <29 weeks’ gestation, are particularly vulnerable to infection due to a combination of a more immature immune system, the intensive care environment, and the invasive interventions necessary for their survival. As such, VPIs are subject to unique immunological challenges.

Understanding the relationship between metabolic adaptation and the maturing immune system in VPIs is further complicated by the limited consensus surrounding neonatal PN guidelines and the wide variations in PN composition and clinical practice. Multi-omics analyses offer an opportunity to approach our understanding of VPI’s development as a system, studying changes in molecular elements globally. Assessment of changes in molecular functions and pathways related to metabolism, immune function, and signalling in an untargeted manner can provide unique and relevant information to shed light on the key stages needed for VPIs to develop adequately. There are major limitations on the availability of clinical biological samples (especially blood) in very preterm infants, with ethical, practical, and financial implications. Nevertheless, these technologies provide the best comprehensive snapshot of the system under study and may provide a framework for understanding to aid the development and optimisation of highly effective PN regimes.

The aim of this pilot exploratory study was to study the feasibility of the application of transcriptomics and metabolomics technologies to discover molecular changes in VPIs over the first 10 days of postnatal adaption (the key PN-dependent period), with sampling at day 3 and day 10 after birth. Specifically, this study will help generate hypotheses that investigate how immune system development may be impacted by differences in parenteral immunonutrient intake, reflecting current controversies in neonatal PN regimen design, such as conditionally essential amino acid content, intravenous lipid emulsion oil source/dose, and trace element provision.

## 2. Methods

### 2.1. Patient Recruitment and Sample Collection

The study received all necessary ethical and regulatory approvals. Very preterm infants born <29 weeks’ gestation and/or <1200 g were eligible for inclusion in the study. A total of 200 µL of whole blood was collected alongside blood for routine biochemistry on days 3 and 10 of life. Immediately following sampling, 600 µL of Blood RNA Buffer (ZR Whole-blood RNA MiniPrep™) was added, as per the manufacturer’s instructions (Zymo Research, Irvine, CA, USA), and the sample was stored at −80 °C. Infants had all other blood samples collected as indicated as part of their routine NICU management, which included full blood counts, electrolytes, and CRP monitoring.

### 2.2. RNA Extraction and Microarray Data Acquisition and Analysis

Samples were thawed in batches for RNA extraction. The extraction was performed as per the manufacturer’s instructions using the ZR Whole-blood RNA MiniPrep™ kit, which included the use of in-column deoxyribonuclease I (DNAse; Sigma Chemical Co., St. Louis, MO, USA). The RNA concentration and quality of the sample was assessed using a Nanodrop spectrophotometer (NanoDrop ND-1000; Thermoscientific, Waltham, MA, USA), and the RNA samples were then aliquoted and stored at −80 °C.

Sixteen RNA samples, free from DNA, were prepared for hybridisation onto Agilent SurePrint G3 Human Expression v3 8 × 60K microarrays (Design ID 072363; Agilent Technologies Inc., Santa Clara, CA, USA) [20]. A total of 100 ng of total RNA was used as input into the Agilent One-Colour Microarray-Based Gene Expression Analysis protocol (Low Input Quick Amp Labelling version 6.9.1), according to the manufacturer’s instructions. A total of 600 ng of Cyanine-3 labelled cRNA was fragmented and loaded onto the arrays, which were hybridised for 17 h at 65 °C, and rotated at 10 rpm in an Agilent hybridisation oven. Following hybridisation, the arrays were washed according to the manufacturer’s instructions and scanned using the Agilent G2505C Microarray Scanner, using the protocol G3_GX_1 colour and 3 µm resolution. The data were extracted using Agilent Feature Extraction software version 12.0.2.2 and its inbuilt data quality assessment resulted in the identification of two samples with low RNA that did not pass quality control. All other RNA samples had a RIN value of >7. This resulted in seven samples each for day 3 and 10 of life, with six paired (day 3 and day 10) samples. Expression data was normalised, logged2 and analysed using Significance Analysis of Microarrays (SAM) [21] within the statistical software R (Version 4.2.0) [22]. Significant genes, defined as 5% FDR (false discovery rate), were used for Principal Component Analysis (PCA, performed on mean-centred and scaled data via singular value decomposition using the function prcomp), heatmap generation, and gene set enrichment analysis using the packages clusterprofiler and ReactomePA [23,24], with parameters minset 10, maxset 200, and 1000 permutations. *p*-values were adjusted for multiple testing via Benjamini–Hochberg method [25], and adjusted pvalues lower than 0.05 were considered significant. All plots were undertaken in R using the package ggplot2 [22,26]. The list of significant genes and respective fold changes was also used for an enrichment in Ingenuity^®^Pathway Analysis™ (IPA) (Qiagen Inc., Germantown, MD, USA), with background set manually to the microarray probe-gene entirety. Analysis was performed according to the manual, and canonical pathways and upstream regulators significantly enriched after *p*-value adjustment via B–H were taken forward for discussion.

### 2.3. H NMR Metabolomics Data Acquisition and Analysis

To remove protein and lipids, serum samples were filtered through a 3000 Da molecular weight cut-off ultracentrifugal filter (Amicon Ultra-0.5 mL, Millipore Sigma, Burlington, MA, USA). To the resulting filtrate, an internal standard solution containing 4.47 mM DSS-d6 (3-(trimethylsilyl)-1-propanesulfonic acid-d6 sodium salt) in 99.8% D_2_O containing 0.2% NaN_3_, was added in a 1:10 (v:v) ratio. Sample pH was adjusted to 6.8 ± 0.1 using small amounts of NaOH or HCl. Final concentrations were corrected for dilution. All ^1^H-NMR spectra were acquired at 25 °C using the first transient of a NOESY pulse sequence on a Bruker Avance 600 MHz spectrometer (Bruker, Billerica, MA, USA) as described by He et al. [27]. All free induction decays were zero-filled to 64,000 data points and spectra were Fourier-transformed using a line broadening of 0.5 Hz, followed by manual phase and baseline correction using Chenomx NMR Suite Processor (version 8.4, Chenomx, Edmonton, AB, Canada). The methyl singlet produced by DSS internal standard was used for chemical shift referencing (set to 0 ppm) and for quantification. The metabolite concentrations for the 14 paired samples were determined using Chenomx NMR Suite Profiler (version 8.4, Chenomx, Edmonton, AB, Canada). A two-sample paired SAM test was applied. Significant metabolites were defined as having an FDR < 5%. These selected metabolites were used to calculate PCA as described above.

### 2.4. Real-Time qPCR

Real-time PCR was used to validate selected microarray results. Samples of RNA were reversed-transcribed to cDNA and used for the validation procedure. Predeveloped TaqMan^®^ gene expression arrays (Applied Biosystems, Foster City, USA) were used to determine the levels of expression of the following genes: April (Hs00601664_g1), Baff (Hs00198106_m1), TLR2 (Hs02621280_s1), TLR4 (Hs00152939_m1), S100A9 (Hs00610058_m1), and STAT3 (Hs00851655_g1). The levels of expression were determined using the relative quantification method with the ΔCt calculation, using L32 (Hs00851655_g1) as the housekeeping gene.

## 3. Results

### 3.1. Patient Recruitment and Characteristics

**Figure 1:**
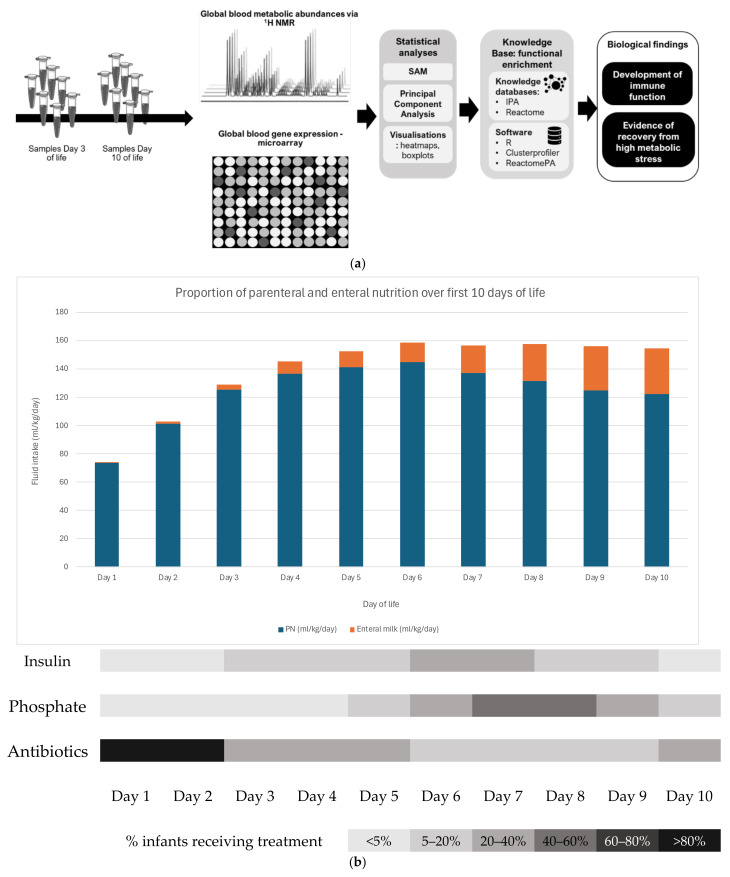
(**a**): Summary illustration of the experimental workflow, data analyses, and key findings. (**b**) Schematic showing the variation in the parenteral and enteral feeds over the first 10 days of life in the study population using mean daily volumes and the % infants (using the greyscale key) receiving treatments that potentially impact immunonutrition strategies: insulin treatment for hyperglycaemia, phosphate supplementation for hypophosphataemia, and antibiotic therapy.

This prospective, exploratory physiological study gained the necessary ethical and regulatory approvals and was conducted at Liverpool Women’s Hospital in the autumn of 2016. Written informed consent was obtained from the parents of all the study participants. Eight infants were recruited with a mean gestational age at birth of 26^+4^ weeks’ (SD: 1.8 weeks) and a mean birthweight of 871 g (SD: 194 g). There were five males and three females in the group. All the mothers had received antenatal steroids, and all infants were inborn at Liverpool Women’s Hospital, Liverpool, UK. Figure 1a summarises the experimental workflow used in this study. Figure 1b is a schematic illustrating PN-dependence in the sample over the 10-day study period. Previously, we have shown that enteral feed volume exceeds parenteral feed volume by day 12 [2].

### 3.2. Omics Analysis

An overview of the experimental design, analysis, and results can be seen in Figure 1a. The holistic nature of these experiments allowed us to detect, in an unbiased way, transcripts and metabolites that change between day 3 and day 10 of life in preterm infants. Using statistical mapping of biological pathways and processes, we have been able to hypothesise some of the underpinning mechanisms involved. The schematic in 1b shows how nutrition sources change over the first 10 days of life, together with the most common metabolic complications, using the daily frequency of insulin-treated hyperglycaemia and additional phosphate supplementation based on previous data in this population [2]. Hyperglycaemia and hypophosphataemia are the most common early metabolic complications in this population. Both are associated with neonatal sepsis [5,8]; the latter may result from effects on cellular energy metabolism and/or membrane synthesis.

**Figure 2:**
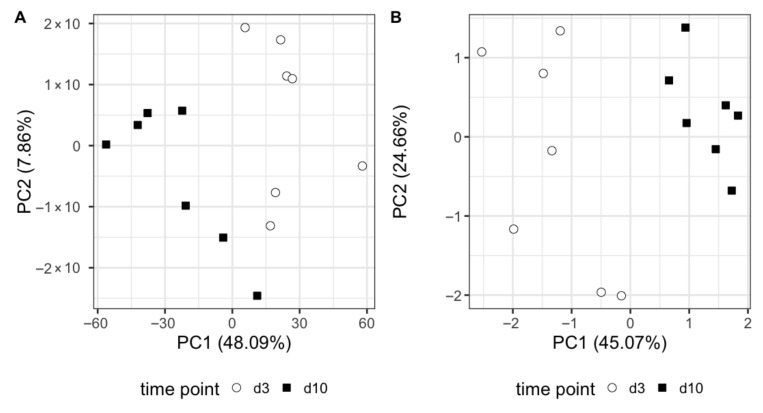
PCA score plots of the first two principal components. Each point represents a sample. (**A**) PCA undertaken with the gene expression values of the 1185 significant genes. (**B**) PCA undertaken with the metabolic abundances of the five significant metabolites. Both PCA plots show excellent discrimination between day 3 (open circles) and day 10 (closed squares) of life, mainly along the x axis.

**Figure 3:**
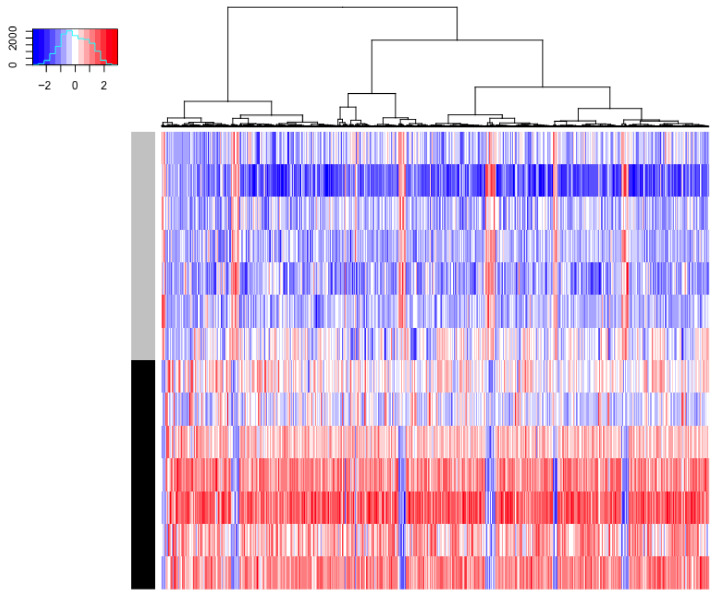
Heatmap representing gene expression of the 1185 significant genes at day 3 samples (grey bar on the left) and day 10 samples (black bar on the left). The range of expression goes from low (blue) to high (red). All genes are clustered by expression using Ward’s hierarchical clustering. Most genes have higher expression in D10 vs. D3.

**Figure 4:**
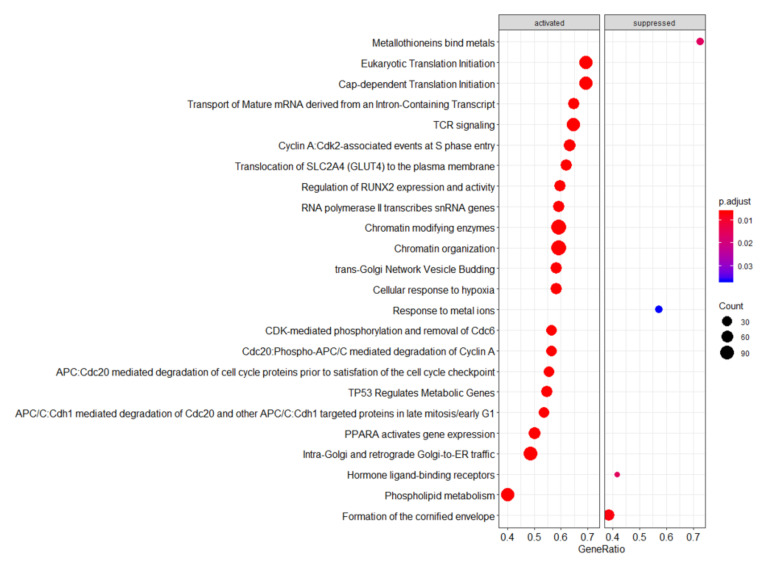
Reactome gene set enrichment analysis results. The panel represents the top 20 significant pathways. The colour of each point represents the significance of enrichment. The size of each point represents the number of genes overlapping in the pathway. The x axis represents the size of the mapped genes with respect to the pathway size.

Gene expression statistical analysis resulted in 1090 genes upregulated and 95 genes downregulated between our two timepoints. We can see at a glance that these genes are able to separate effectively both sample types, as shown in the PCA score plot in Figure 2A and the heatmap in Figure 3. A full list of these genes, expression means, and statistics is provided in Supplementary Table S1. It is noticeable that the vast majority of genes were upregulated (red colour) between days 3 and 10 of life. We used these gene lists to run gene set enrichment analyses in two databases: Reactome and IPA. Reactome results can be seen in Figure 4. While not many genes are downregulated, we see that metallothioneins and response to metal ions are suppressed, as well as hormone ligand-binding receptors and formation of the cornified envelope. The rest of the pathways are activated and can mainly be grouped in immune function (such as TCR signalling), overall developmental processes (such as regulation of runx2 expression, which is involved in osteogenesis), and metabolism (such as phospholipid metabolism). IPA revealed three significantly enriched pathways with a positive Z-score: fMLP signalling in neutrophils, Fcγ receptor-mediated phagocytosis in macrophages and monocytes, and phospholipase C signalling. All these pathways are active in immune system development and cellular responses.

**Figure 5:**
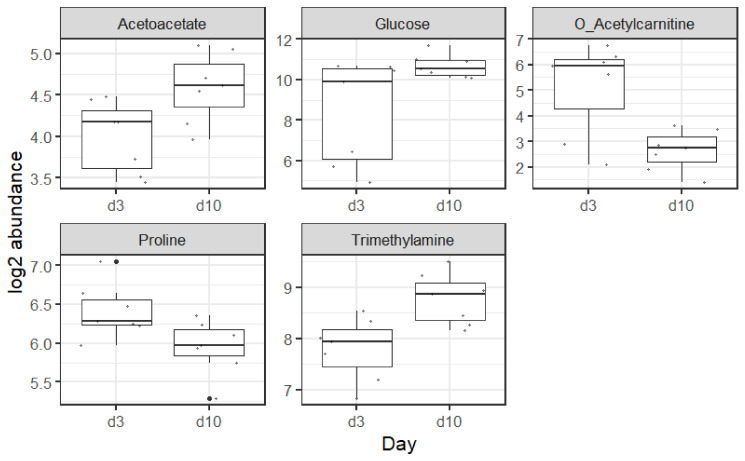
Significant metabolites between day 3 and day 10 of life. Boxplots represent the sample distribution (first and third quantiles as well as the median). Overlay points represent the sample values.

^1^H NMR metabolomics analysis revealed five metabolites with significant differences. These are acetoacetate, glucose, o-acetylcarnitine, proline, and trimethylamine. These five metabolites are enough to fully discriminate samples at day 3 and day 10 of life, as shown in the PCA in Figure 2B. Boxplots of these metabolites are shown in Figure 5.

### 3.3. Real-Time qPCR Validation

The results obtained from qPCR validation experiments were consistent with the results of the microarray analysis, and no significant differences were found between the results obtained using the two different techniques.

### 3.4. Results Summary

**Figure 6:**
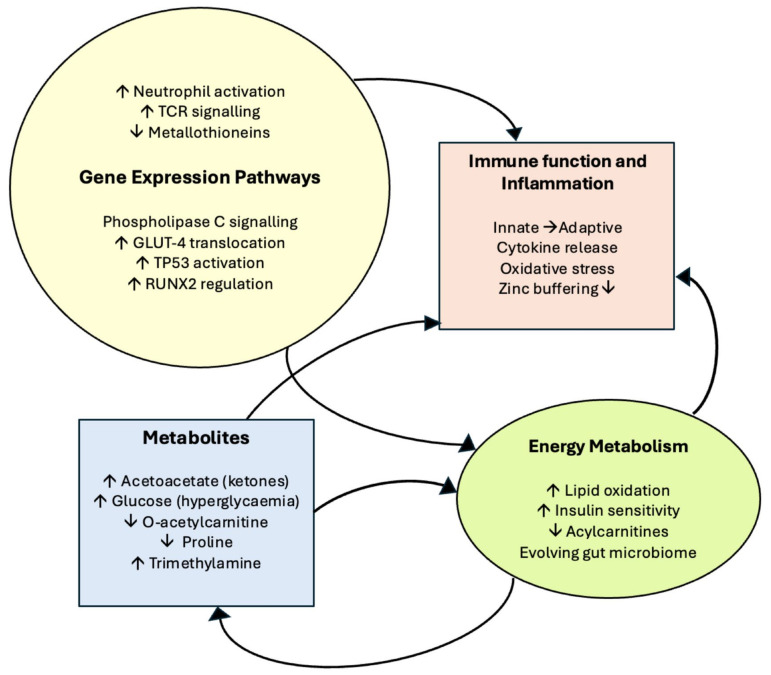
Simple schematic summarising the study findings in relation to key aspects of cellular function, immune function, and metabolism.

A schematic summarising the main study findings grouped together in relation to key aspects of cellular function, immune function, and metabolism is shown in Figure 6.

## 4. Discussion

The analysis presented here emphasises the multi-perspective view that multi-omics analysis offers during the critical period of postnatal adaptation. The approach to statistical and pathway analysis has ensured that reporting of significant pathways is robust. It is noteworthy that IPA identified only immune-related pathways, discussed in more detail below. These suggest the early days of life as a critical period of immune system development and metabolic changes in the very preterm infant. Processes related to neutrophil activation, phagocytosis by monocytes and macrophages, and T-cell activation are all shown to be upregulated. Reactome pathway analysis identified a wider range of processes upregulated in the postnatal adaptation in the first 10 days of life at the molecular level, including those associated with energy and carbon metabolism, protein turnover, cell signalling, cell cycle, and DNA and RNA repair, all of which tie in with the expected developmental changes following birth. The apparent downregulation of metallothionein metabolism is discussed further below but illustrates how transcriptomics can identify changes in metabolic pathways with implications for immune adaptation. In contrast, metabolomics describes the biochemical environment, heavily influenced by nutritional strategy in the preterm population. This helps generate hypotheses linking adverse metabolic complications that are associated with impaired immune response such as hyperglycaemia or hyperlipidaemia. Understanding factors determining metabolic resilience in the face of sepsis or other inflammatory responses is an important concept in improving outcomes in high-risk populations like preterm infants [28].

### 4.1. Ingenuity Pathway Analysis (IPA)—Significantly Upregulated Pathways

#### 4.1.1. fMLP Signalling in Neutrophils

Activation of the fMLP signalling pathway in neutrophils results in neutrophil activation, migration, increased phagocytosis, and cytokine release [29]. This observed up-regulated expression of genes associated with the neutrophil response to the bacteria product fMLP is consistent with an increase in the functional maturity of neutrophils in these infants between three and ten days after birth and raises the possibility that these cells could already be responding to the presence of bacterial pathogens [30].

#### 4.1.2. Fcγ Receptor-Mediated Phagocytosis by Macrophages and Monocytes

Binding of IgG-coated targets such as immune complexes or opsonized pathogens through macrophage and monocyte Fc receptors plays an important role in the immune response to pathogens, not only by leading to pathogen phagocytosis and removal, but also by stimulating further responses [31]. Here, it would be dependent on maternal antibody but again indicates further evidence for the ongoing development of a functional immune system and indicates that our transcriptional analysis is able to detect significant changes in these infants after birth. The upregulation of this pathway by day 10 of life adds further evidence to the ongoing development of a functional immune system.

#### 4.1.3. Phospholipase C Signalling

Phospholipase C (PLC) is a lipid-metabolising enzyme and plays a significant role in transmembrane signalling. Uniquely, it can produce three distinct signals, all of which are involved in the regulation of ion channels, including calcium ion channels. Activation of PLC signalling occurs early following binding of various agonists to cell-surface receptors, and it is involved in a diverse range of processes [32]. Its upregulation by day 10 on the gene set enrichment profile is in some ways unsurprising, given its involvement in a vast array of pathways and the rapid developmental changes occurring in the preterm infant.

### 4.2. Reactome—Highlighted Significant Pathways

Upregulation of TCR can be associated with a move from an unresponsive state comparable to that seen in immature T-cells towards a more fully competent cell able to make a response. Non-immune specific pathways indicated to be activated included those associated with energy and carbon metabolism, protein turnover, cell signalling, cell cycle, and DNA and RNA repair, all of which tie in with the expected developmental changes following birth.

Fewer pathways were indicated to be downregulated, the most striking being metallothionein activity, indicated by Reactome to be the most significantly reduced. Interestingly, metallothionein expression is induced by oxidants, toxins, and electrophiles. By neutralising reactive oxygen species, metallothioneins protect blood cells from oxidative stress-induced cytotoxicity [33]. Moreover, they play a role in the regulation of zinc levels. Very low birthweight babies are at risk of hypoxic brain damage, and its severity is influenced by the activity of zinc-dependent metallothioneins [34]. It can be hypothesised that during the first days of life, expression of metallothioneins is critical for the immediate postnatal adaptation, and as the infant adjusts to the new environment, a pertinent downregulation is seen. Low zinc levels in early neonatal life are recognised as a potential cause of immune dysfunction, and there is evidence of an inverse relationship between early zinc levels and gestation [35]. Neonatal primate data suggest that metallothionein levels are low immediately after birth [36]. Downregulation of pathways involved in metallothionein may be an important mechanism to ensure adequate intracellular free zinc is available for optimal immune function [37]. Similarly, downregulation of metallothioneins has also been described in intestinal samples from infants with necrotising enterocolitis, suggesting it may have complex roles in inflammatory processes [38].

### 4.3. Metabolites

The five metabolites that differentiated the day 3 from the day 10 samples were acetoacetate, glucose, o-acetylcarnitine, proline, and trimethylamine.

#### 4.3.1. Acetoacetate

Acetoacetate is a ketone body responsible for energy provision in low-carbohydrate states. Preterm infants have been shown to have low concentrations of ketone bodies throughout the first week of life, with no rise in ketone body concentration in response to low blood glucose levels, as would be the expected response in term infants [39]. This impaired ketogenesis in preterm infants is further supported by low basal levels of acetoacetate in preterm infants and no change in their levels in response to feeding [40]. It can be postulated that the higher levels of acetoacetate on day 10 of life in our study reflects a maturation of this process with the ability to generate ketone bodies. There is also the potential that insulin insensitivity results in decreased glucose uptake into cells, despite higher glucose levels, and therefore an ongoing reliance on non-carbohydrate metabolism. Nilsson et al. [41] found preterm infant ketone levels on day one of life to be the highest in infants born <26 weeks. The increase in acetoacetate observed on day 7 and 14 of life, especially in the lower GAs, was thought likely to reflect a high intake of parenteral lipids and increased plasma lipid levels [41]. This sharp rise in acetoacetate early postnatal period then falls back as the transition to enteral feeding takes place.

#### 4.3.2. Glucose

The higher levels of glucose on day 10 of life is unsurprising, given that hyperglycaemia in extremely preterm infants is well recognised, with a pattern that shows a peak in glucose levels at the end of the first week of life [42]. There are many physiological components involved in preterm hyperglycaemia including exogenous glucose load, persistent inappropriate endogenous glucose production, stress response, reduced insulin production secondary to β-cell repopulation, immaturity, and insulin insensitivity. One of the underlying mechanisms that have been proposed is immature expression of glucose transporters alongside limited adipose and skeletal muscle mass [43]. The gene set enrichment results indicate upregulation of glucose transporter 4 (GLUT 4) to the plasma membrane (where it can transport glucose intracellularly) [44] by day 10 of life. It is possible that this occurs in response to insulin stimulation, reflecting the maturation of insulin sensitivity occurring in the infant’s second week.

#### 4.3.3. O-Acetylcarnitine

Raised plasma acylcarnitine levels, including free carnitine [45] and acetylcarnitine [46], in the first three days following preterm birth are associated with pre-eclampsia. Falling levels are likely to reflect the end of transplacental supply from the mother. In addition, very preterm infants are often in a state of catabolism in the first days of life following removal of the placental nutritional supply, their lack of energy stores, and slow tolerance of feeds. The metabolite o-acetylcarnitine, involved in fatty acid oxidation and cellular energy metabolism, was found in higher levels on day 3 of life, with lower levels on day 10, likely reflecting the improved nutritional status of these infants by this timepoint with a reduced requirement for non-carbohydrate-based metabolism. It has been reported elsewhere that metabolomic profiling of extremely preterm infants clusters by postnatal day at sampling, primarily driven by shifts in acylcarnitine concentrations with reductions over time, as was seen in our study [47]. It is known that preterm infants are subject to high levels of oxidative stress from exposure to higher partial pressures of oxygen postnatally, in addition to their immature systems and the required medical interventions [48]. It has been postulated that acetylcarnitine is a moderator of oxidative stress and involved in redox homeostasis, and the lower levels on day 10 of life in our cohort may reflect a reduction in their oxidative stress by day 10 of life [49].

#### 4.3.4. Proline

Proline is a conditionally essential amino acid, and it is a precursor for arginine synthesis (another conditionally essential amino acid) in enterally fed preterm neonates [50]. It is known that very preterm infants often have low plasma levels of conditionally essential amino acids during their period of PN dependency [51] A fall in proline over the early postnatal period in very preterm infants has been described by Nilsson et al. [41]. This may reflect improved arginine status between day 3 and day 10. Preterm infants are arginine-deficient and proline (not glutamate) is the preferred substrate for enterally fed preterm infants [50] as enteral feeds increase between day 3 and 10. In our own published plasma AA data, we have noted the inverse relationship between arginine and proline in the first 10 days of life [51].

#### 4.3.5. Trimethylamine

Trimethylamine is produced by the breakdown of dietary quaternary amines, such as choline, by the gut microbiota. Free choline is found in high concentrations in human milk, and it is possible the higher levels of trimethylamine on our day 10 of life samples reflect both the increased colonisation and establishment of the gut microbiome alongside the increasing volumes of enteral human milk that the infants are consuming by day 10 of life in comparison to day 3 [52].

There are several groups who have reported trimethylamine involvement in the development of metabolic and cardiovascular diseases, and higher levels have been found in the metabolomic profiles of ex-preterm adults with aberrant adiposity [53,54].

### 4.4. Limitations of the Study

This was a small exploratory physiological study and, as such, has limitations due to the small number of infants studied, over a relatively short period, and the inherent heterogeneity of patient phenotype and clinical course within such a population. This approach was needed to test the methodology that combines longitudinal analysis with multi-omics analysis of blood or plasma. The volume of additional blood that can been taken from very preterm infants in the first 28 days severely limits the volume and frequency of each individual sample. We have confirmed that it is feasible to use multi-omics analysis at two timepoints in the first 28 days of life. These timepoints reflect both a critical period of metabolic and immune adaptation and exposure to a specific nutritional environment (PN dependency). We have previously shown in our population of very preterm infants that the maximum PN intake is between day 3 and day 10. The transition to full milk feeds is complete by day 14 in >80% of infants, and therefore the metabolic environment is likely to change significantly. There is good evidence for this in the other study to examine preterm longitudinal metabolomic data [41]. Despite the small sample size, our data were able to identify the same changes in key metabolites described in this larger study. Future study design would include further longitudinal data, for example, on day 30 and 36 weeks corrected gestational age. This would help distinguish between transient and more permanent changes following differences in early parenteral immunonutrient exposure. Future studies would require a sample size large enough to investigate potentially important co-variates, such as gestation, birthweight, sex, and macronutrient intake. This pilot study was not large enough to investigate these co-variates.

The transcriptional analysis conducted in this study provides significant insight into possible functional changes in metabolism and gene expression in preterm infants. However, further studies together with the development of practical methods using the small samples available in this patient group, are necessary to confirm whether the potential changes observed at the transcriptional level correlate with actual functional changes. All the participants’ mothers had received antenatal steroids, which are thought to influence gene expression in preterm infants [55]; however, as this is standard care in cases of suspected preterm delivery, it is likely to reflect the experience of other neonatal populations in high-income countries.

### 4.5. Future Work

We have shown that the concept of multi-omics can be applied to extremely preterm infants in a clinical setting during the critical period of postnatal adaptation. Understanding adaptive processes is a critical step in precision design of immunonutrient interventions (such as amino acid or trace element supplementation) for future randomised controlled trials in this challenging population. Moreover, this approach recognises that such interventions are dependent on different biologic vulnerabilities and subsequent health trajectories, not just on gestation, birthweight, or sex [56]. Furthermore, it is increasingly recognised that disease categories are oversimplified and that those previously considered as a single entity may have multiple aetiologies, with different mechanisms, biomarkers, and responses to treatment. Necrotising enterocolitis is a notable example [57], where multi-omics may enable a more targeted approach to immunonutrient interventions. Other important neonatal morbidities, such as sepsis [58] and bronchopulmonary dysplasia [59], could also benefit from this approach.

## 5. Conclusions

This study confirms the feasibility of multi-omics analysis in very low birthweight babies enabling insight into postnatal developmental transformations that occur in these infants. It provides direction for future research and highlights critical processes in post-natal adaption in the VPIs. Furthermore, we shed light on a potential list of markers to be used to assess infant development under different conditions, linking these markers to known events related to metabolic and immune development.

## Data Availability

The original contributions presented in this study in relation to transcriptomics are included in the Supplementary Material. Requests to access the datasets should be directed to the corresponding author.

## Supplementary Materials

The following supporting information can be downloaded at: https://www.mdpi.com/article/10.3390/metabo15100659/s1, Table S1: Full DEG lists with gene symbols, expression means and statistics.

## Abbreviations

PN	Parenteral nutrition
GALT	Gut-associated lymphoid tissue
VPI	Very preterm infant
µl	Microlitre
RNA	Ribonucleic acid
NICU	Neonatal intensive care unit
CRP	C-reactive protein
DNAse	Deoxyribonuclease
DNA	Deoxyribonucleic acid
ng	Nanogram
rpm	Revolutions per minute
µm	Micrometre
RIN	RNA integrity number
SAM	Significance Analysis of Microarrays
FDR	False discovery rate
PCA	Principal Component Analysis
IPA	Ingenuity^®^Pathway Analysis™
B–H	Benjamini–Hochberg
NMR	Nuclear magnetic resonance
Hz	Hertz
DSS	Sodium 3-(trimethysilyl)propane-1-1sulfonate
ppm	Parts per million
PCR	Polymerase chain reaction
cDNA	Complementary deoxyribonucleic acid
Ct	Cycle threshold
SD	Standard deviation
g	Grams
TCR	T-cell receptor
fMLP	N-Formylmethionyl-leucyl-phenylalanine
PC	Principal component
d3	Day 3
d10	Day 10
IgG	Immunoglobulin G
PLC	Phospholipase C
GA	Gestational age
GLUT 4	Glucose transporter 4
